# Bridging the Gap Between Experimental and Clinical Applications of Mesenchymal Stromal Cell Therapy in Intervertebral Disc Regeneration

**DOI:** 10.3390/bioengineering13101234

**Published:** 2026-10-21

**Authors:** Lidija Gradisnik, Borut Prestor, Matic Munda, Tilen Zele, Uros Maver, Nina Kocivnik, Tomaz Velnar

**Affiliations:** 1 Institute of Biomedical Sciences, Medical Faculty of Maribor, 2000 Maribor, Slovenia; lidija.gradisnik@um.si (L.G.); uros.maver@um.si (U.M.); nina.kocivnik@um.si (N.K.); 2 Department of Neurosurgery, University Medical Centre Ljubljana, 1000 Ljubljana, Slovenia; borut.prestor@kclj.si (B.P.); matic.munda@kclj.si (M.M.); tilen.zele@kclj.si (T.Z.); 3 Medical Faculty of Ljubljana, 1000 Ljubljana, Slovenia; 4 Alma Mater Europaea University Maribor, 2000 Maribor, Slovenia

**Keywords:** intervertebral disc, regenerative medicine, mesenchymal stromal cells, biomarkers, tissue engineering

## Abstract

Intervertebral disc (IVD) degeneration is a major cause of chronic low back pain and disability worldwide. Although current treatment strategies effectively alleviate symptoms, they do not restore disc structure or reverse the underlying biological processes responsible for degeneration. Mesenchymal stromal cell (MSC)-based therapies have emerged as promising regenerative approaches owing to their immunomodulatory, anti-inflammatory, and extracellular matrix-regenerative properties, mediated predominantly through paracrine signalling rather than long-term cellular engraftment. Despite encouraging preclinical results, clinical translation remains limited by poor cell survival within the hostile disc microenvironment, protocol heterogeneity, and insufficient long-term clinical evidence. This review critically examines the current state of MSC-based therapy for IVD degeneration by integrating experimental findings, clinical evidence, and emerging translational strategies. Particular emphasis is placed on the biological mechanisms of MSC-mediated regeneration, comparison of different MSC sources, advances in extracellular vesicle- and exosome-based cell-free therapies, and the growing role of quantitative magnetic resonance imaging, molecular biomarkers, and precision diagnostics in patient selection and treatment monitoring. In addition, the review discusses the major biological, technical, manufacturing, and regulatory challenges that currently limit clinical implementation. Collectively, the evidence indicates that successful translation of MSC-based therapies will require not only optimised cell products but also standardised manufacturing, biomaterial-assisted delivery systems, advanced diagnostic biomarkers, and well-designed multicentre clinical trials. Integrating these advances will be essential for transforming MSC-based therapies into safe, standardised, and disease-modifying treatments for patients with intervertebral disc degeneration.

## 1. Introduction

Intervertebral disc (IVD) degeneration is a leading cause of chronic low back pain and disability worldwide, representing a substantial socioeconomic burden [1,2]. The degenerative process involves progressive extracellular matrix degradation, depletion of nucleus pulposus cells, chronic inflammation, and structural deterioration, resulting in impaired biomechanical function and pain [2]. Current therapeutic approaches, including pharmacological treatment, physiotherapy, and surgical interventions, primarily focus on alleviating symptoms and maintaining spinal stability rather than promoting disc regeneration. As a result, these treatments do not directly address or reverse the underlying biological mechanisms driving disc degeneration.

Regenerative medicine has emerged as a promising approach for treating IVD degeneration. Mesenchymal stromal cells (MSCs) have attracted particular interest because of their ability to modulate inflammation, stimulate extracellular matrix synthesis, and support tissue repair through differentiation and paracrine signalling [3,4]. In accordance with the International Society for Cell & Gene Therapy (ISCT) recommendations, the term ‘mesenchymal stromal cells’ is used throughout this review to describe heterogeneous, culture-expanded cell populations investigated in regenerative applications [5]. The term ‘mesenchymal stem cells’ should more specifically refer to populations with rigorously demonstrated stem-cell properties, including self-renewal and differentiation capacity.

MSCs can be isolated from various tissues, including bone marrow, adipose tissue, and umbilical cord, and have demonstrated promising effects in experimental models of disc degeneration. Increasing evidence suggests that their therapeutic activity is largely mediated by secreted bioactive molecules and extracellular vesicles rather than long-term engraftment [6]. Nevertheless, clinical translation remains challenging. The hostile microenvironment of the degenerated disc, characterised by hypoxia, nutrient deprivation, acidic pH, hyperosmolarity, and chronic inflammation, limits MSC survival and regenerative efficacy. Moreover, heterogeneity in cell sources, manufacturing protocols, delivery methods, patient selection, and outcome measures complicates comparisons between studies and contributes to inconsistent clinical results [7,8,9]. Although early clinical trials have reported encouraging safety profiles and improvements in pain and function, evidence of durable structural regeneration remains limited. Advances in biomaterial-assisted delivery, genetic engineering, extracellular vesicle-based therapies, imaging, and molecular biomarkers may help address these limitations. Greater emphasis on standardisation, Good Manufacturing Practice (GMP)-compliant production, objective biomarkers, and multicentre clinical trials is also essential for successful clinical translation [6,7,8,9].

This review examines the evidence regarding MSC-based therapies for IVD degeneration, focusing on the translational gap between experimental and clinical studies. While previous reviews have addressed individual aspects of MSC-based regeneration, including therapeutic mechanisms, cell sources, extracellular vesicles, biomaterials, and clinical outcomes, the present review integrates these developments with the diagnostic, manufacturing, and regulatory requirements for clinical implementation [4,6,7,8,9]. Particular emphasis is placed on connecting advances in MSC biology and cell-free therapies with quantitative imaging and molecular biomarkers for patient selection, treatment monitoring, and evaluation of therapeutic response. Through this integrated translational perspective, the review aims to identify the key steps required to bridge the gap between experimental findings and standardised, precision-oriented clinical applications of MSC-based therapies for IVD degeneration [8,9].

### Literature Search Strategy

A literature search was conducted using the PubMed and Scopus databases to identify relevant publications on mesenchymal stromal cell-based approaches and therapies for intervertebral disc degeneration and regeneration. The search was restricted to studies published between 2015 and 2026 to ensure that the review reflects contemporary scientific knowledge, recent technological advances, and current perspectives on clinical translation. The following keywords were used individually and in various combinations: “intervertebral disc”, “intervertebral disc regeneration”, “regenerative medicine”, “mesenchymal stromal cells”, “mesenchymal stem cells”, “stem cell therapy”, “paracrine signalling”, “immunomodulation”, “extracellular vesicles”, “exosomes”, “cell priming”, “genetic engineering”, “3D bioprinting”, “biomaterials”, “biomarkers”, “tissue engineering”, and “clinical application”. Studies were considered for inclusion if they addressed experimental or clinical aspects of MSC-based therapies for intervertebral disc degeneration and regeneration, including regenerative mechanisms, cell-based and cell-free therapies, advanced tissue-engineering approaches, diagnostic biomarkers, and clinical translation. Original research articles, preclinical and clinical studies, and relevant review articles were considered. Publications outside the defined period, studies not directly related to intervertebral disc regeneration or MSC-based regenerative approaches, and articles outside the scope of the review were excluded. As this article is a narrative review, studies were selected based on their scientific relevance and contribution to the principal topics addressed rather than according to a formal systematic-review protocol. Particular emphasis was placed on contemporary evidence concerning MSC-mediated paracrine signalling, immunomodulation, extracellular vesicle-based therapies, advanced biomaterial-assisted delivery strategies, and the challenges associated with translating experimental findings into clinical practice.

## 2. Overview of Intervertebral Disc Degeneration

Intervertebral disc degeneration begins with a gradual reduction in the number of large notochordal cells within the nucleus pulposus, disrupting disc homeostasis. This initiates a degenerative cascade characterised by extracellular matrix degradation, increased matrix metalloproteinase (MMP) activity, a shift from type II to type I collagen synthesis, and proteoglycan depletion [10,11]. Consequently, the disc progressively loses its water-binding capacity, resulting in dehydration, reduced turgor pressure, and structural deterioration. These changes compromise spinal mechanical stability and increase susceptibility to repetitive microtrauma [10].

As degeneration progresses, nucleus pulposus cells produce increased levels of pro-inflammatory and catabolic cytokines, including IL-1 (interleukin-1), IL-6 (interleukin-6), IL-12 (interleukin-12), IL-17 (interleukin-17), TNF-α (tumour necrosis factor-α), IFN-β (interferon-β), and IFN-γ (interferon-γ), further intensifying degeneration [1,2]. These mediators promote angiogenesis, granulation tissue formation, fibrosis, and activation of nociceptive nerve endings. Additional contributing factors include ischaemia, excessive mechanical stress, increased catabolic enzyme activity, and reduced aggrecan content, which further promote matrix destruction and cellular dysfunction. Sensory nerve fibre ingrowth may also contribute to abnormal disc innervation and discogenic pain [12,13,14].

Ageing is an important contributor to IVD degeneration, associated with changes in disc cellularity, extracellular matrix homeostasis, nutrient transport, and cellular stress responses. A key age-related mechanism is the accumulation of senescent disc cells, characterised by reduced regenerative capacity and a senescence-associated secretory phenotype (SASP) involving increased secretion of pro-inflammatory cytokines and matrix-degrading factors. Together with oxidative stress, impaired cellular metabolism, and changes in the cartilaginous endplates, these processes accelerate extracellular matrix degradation and diminish the intrinsic repair capacity of the disc. Thus, age-related degeneration involves both structural deterioration and alterations in the biological microenvironment that may influence responses to regenerative therapies [15,16].

Advanced degeneration may result in disc bulging, herniation, and nerve root compression, causing pain and neurological symptoms. Altered biomechanics and chronic overload may also promote osteophyte formation, contributing to spinal canal narrowing and impaired spinal function [12,13].

## 3. Clinical Burden and Limitations of Current Therapies

From a clinical perspective, low back pain is the most common symptom associated with degenerative spinal disease and is one of the leading causes of disability worldwide. Current treatment strategies are primarily aimed at symptom control rather than reversing the underlying degenerative process. Conservative management typically includes analgesic or anti-inflammatory medication, physiotherapy, acupuncture, local nerve blocks, and lifestyle modification [10]. If conservative treatment fails to provide adequate relief, surgical options such as microdiscectomy, artificial disc replacement, or spinal fusion may be considered. In more severe cases, laminectomy with or without fusion is performed to decompress the spinal canal and reduce nerve compression. Although surgery may relieve pain and improve functional status in selected patients, it does not directly address the biological mechanisms responsible for disc degeneration. Furthermore, surgical procedures carry risks of operative and postoperative complications, and their long-term outcomes are variable. Post-surgical changes in spinal biomechanics may also accelerate degeneration in adjacent spinal segments, increasing the likelihood of recurrent symptoms and further procedures [1,2,10,11].

## 4. Innovative Approaches to Disc Regeneration

Improved understanding of the molecular and cellular basis of disc degeneration has generated increasing interest in regenerative medicine. Unlike conventional approaches, regenerative therapies aim to restore disc structure and metabolic homeostasis by addressing fundamental pathological processes such as inflammation, matrix degradation, and cellular depletion [1,2]. Among these strategies, stromal cell therapy has attracted particular attention as a potential means of promoting disc regeneration. Experimental studies and clinical trials are currently investigating intradiscal stromal cell implantation into degenerated discs as a treatment for low back pain. The aim of this approach is not only pain relief, but also tissue regeneration and functional restoration of the disc [2,10,11,17]. Because MSCs combine limited differentiation potential with strong trophic and immunomodulatory activity, they are promising candidates for restoring intervertebral disc homeostasis [11,17]. In addition to differentiating into disc-like cells, they can release anti-inflammatory and trophic mediators that support extracellular matrix synthesis and modify the degenerative microenvironment. Preclinical research and early human studies have produced encouraging results, including reductions in pain, improvements in function, and, in some cases, radiological evidence suggestive of disc regeneration [11,12,17].

Autologous stromal cell transplantation, in which cells are obtained from the patient and then reinjected into the disc, reduces the risk of immune rejection and disease transmission. Furthermore, as the intervention is minimally invasive, it may be associated with less postoperative pain, shorter recovery, and fewer complications than conventional surgery [17]. Although current evidence suggests that intradiscal MSC therapy is technically feasible and has an acceptable short-term safety profile, the durability of its therapeutic effects remains uncertain. Nevertheless, by targeting the biological basis of degeneration rather than merely suppressing symptoms, stromal cell-based treatment may represent a promising disease-modifying option for degenerative disc disease. Continued investigation is necessary to determine its long-term effectiveness and clinical utility [11,18,19,20,21].

## 5. Diagnostic Biomarkers for Patient Selection and Monitoring of MSC Therapy

**Figure 1:**
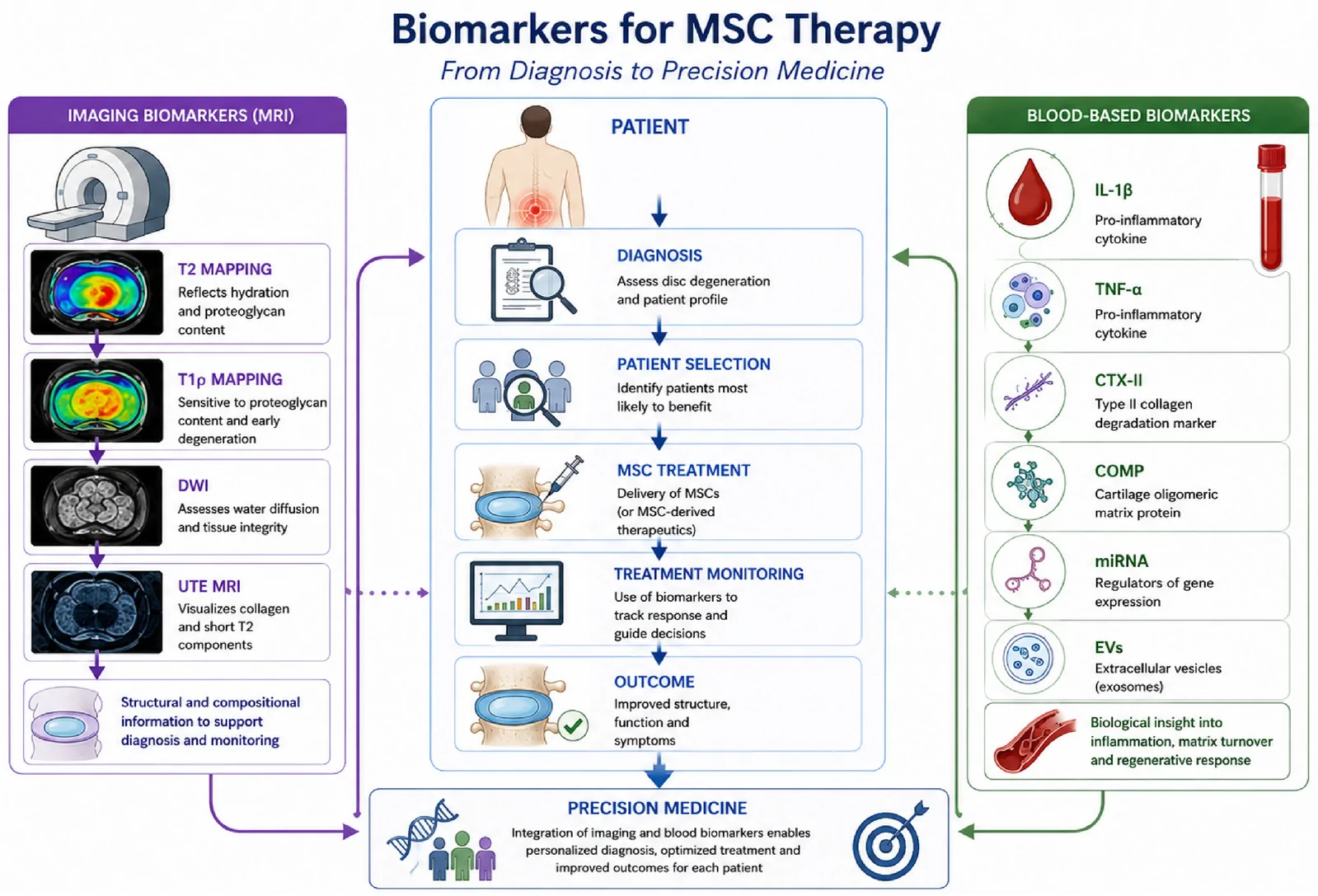
Biomarkers supporting MSC-based therapy for intervertebral disc degeneration. Imaging and blood-based biomarkers inform each stage of the clinical workflow, from diagnosis and patient selection to treatment monitoring and outcome assessment. Quantitative MRI techniques, including T2 mapping, T1ρ mapping, diffusion-weighted imaging (DWI), and ultrashort echo time (UTE) MRI, provide objective information on disc composition, hydration, extracellular matrix integrity, and endplate structure. Blood-based biomarkers, including IL-1β, TNF-α, CTX-II, COMP, microRNAs (miRNAs), and extracellular vesicles (EVs), reflect inflammatory activity, extracellular matrix turnover, and regenerative responses. Integrating these complementary biomarkers enables more accurate patient stratification, objective evaluation of therapeutic efficacy, and supports the development of precision medicine approaches for MSC-based treatment of intervertebral disc degeneration.

Successful clinical translation of MSC-based therapies for IVD degeneration depends not only on optimisation of stromal cell products and delivery strategies but also on the availability of reliable diagnostic biomarkers for patient selection and objective monitoring of treatment efficacy [22,23,24]. Currently, most clinical trials evaluating MSC therapy rely primarily on patient-reported outcome measures such as the Visual Analogue Scale (VAS), Oswestry Disability Index (ODI), and quality-of-life questionnaires. Although these outcomes are clinically relevant, they provide only indirect evidence of tissue regeneration and correlate poorly with the underlying biological status of the intervertebral disc. Consequently, increasing attention has been directed towards imaging, biochemical, and molecular biomarkers that can objectively characterise disc degeneration, identify patients most likely to benefit from regenerative therapies, and monitor biological responses following treatment. Recent advances in quantitative (MRI), including T2 mapping, T1ρ mapping, diffusion-weighted imaging (DWI), and ultrashort echo time (UTE) MRI, together with emerging molecular biomarkers, have substantially improved the objective assessment of disc composition and degeneration, providing promising tools for precision regenerative medicine [22,25]. The diagnostic workflow supporting MSC-based therapy, including imaging and molecular biomarkers for patient selection and treatment monitoring, is summarised in Figure 1.

Despite their potential, the clinical utility of these biomarkers and precision-medicine approaches remains insufficiently established. Much of the available evidence is derived from preclinical studies, exploratory clinical cohorts, or relatively small observational studies, and few candidate biomarkers have undergone independent validation in large, prospective patient populations. Quantitative imaging and molecular biomarkers should therefore currently be regarded primarily as promising research and investigational tools rather than established criteria for routine patient selection or treatment monitoring. Further prospective studies and standardised validation are required before their integration into clinical decision-making [22,23,24].

### 5.1. Advanced MRI Biomarkers

MRI remains the cornerstone for diagnosing intervertebral disc degeneration. Conventional T2-weighted MRI and the Pfirrmann grading system provide valuable information on disc morphology and hydration but primarily detect relatively advanced structural degeneration. Quantitative MRI techniques have therefore emerged as promising biomarkers capable of identifying early biochemical alterations before irreversible structural changes occur [2,22,24]. Among these, T2 mapping has become one of the most extensively investigated imaging biomarkers. T2 relaxation times correlate with water content, collagen organisation, and proteoglycan concentration within the nucleus pulposus. Progressive reductions in T2 values reflect extracellular matrix degradation and loss of disc hydration, making T2 mapping particularly suitable for longitudinal monitoring of regenerative therapies aimed at restoring disc composition [2]. Similarly, T1ρ (T1-rho) mapping is highly sensitive to proteoglycan depletion, one of the earliest biochemical events during IVD degeneration. Several studies have demonstrated that T1ρ values correlate more closely with extracellular matrix integrity than conventional MRI and may therefore detect regenerative changes before alterations become visible on standard imaging [2,22,25].

Additional quantitative MRI techniques, including DWI, diffusion tensor imaging (DTI), and UTE MRI, provide complementary information on tissue microstructure, water diffusion, collagen organisation, and cartilaginous endplate integrity. These modalities have shown considerable potential for evaluating early degeneration and monitoring tissue regeneration following biological interventions, although further clinical validation remains necessary [2,24,25,26].

### 5.2. Biochemical Biomarkers

Degeneration of the intervertebral disc is accompanied by profound changes in inflammatory signalling and extracellular matrix turnover. Consequently, several circulating and tissue-derived biochemical markers have been investigated as potential diagnostic and prognostic biomarkers. Among inflammatory mediators, IL-1β and TNF-α play central roles in the pathogenesis of IVD degeneration. These cytokines promote the production of matrix metalloproteinases, inhibit extracellular matrix synthesis, induce apoptosis in nucleus pulposus cells, and sustain inflammatory processes within the intervertebral disc. Increased levels of both cytokines have been consistently linked to the progression of disc degeneration, suggesting their potential utility as biomarkers for identifying patients with active inflammatory changes who may benefit from the immunomodulatory effects of MSC-based therapies [4,13,14,15,16]. Extracellular matrix degradation can also be assessed using biomarkers such as MMPs, C-terminal telopeptide of type II collagen (CTX-II), cartilage oligomeric matrix protein (COMP), and aggrecan degradation fragments. Increased circulating or tissue levels of these molecules reflect accelerated matrix catabolism and correlate with disease severity. Conversely, reductions following regenerative therapy may provide objective evidence of biological repair. Although these biomarkers have not yet been incorporated into routine clinical practice, they represent promising surrogate endpoints for future regenerative clinical trials [1,26].

### 5.3. Molecular Biomarkers

Recent advances in molecular biology have considerably expanded the range of candidate biomarkers available for precision regenerative medicine in IVD degeneration. Among these, microRNAs (miRNAs) have emerged as key post-transcriptional regulators of inflammation, extracellular matrix (ECM) metabolism, apoptosis, oxidative stress, cellular senescence, and tissue homeostasis [26]. Aberrant expression of numerous miRNAs has been associated with the onset and progression of IVD degeneration, suggesting their potential utility as diagnostic, prognostic, and treatment-monitoring biomarkers. Furthermore, specific miRNA signatures may help identify patients who are most likely to benefit from regenerative therapies, including MSC-based interventions. Growing attention has also been directed towards extracellular vesicles (EVs), particularly MSC-derived exosomes, as both therapeutic agents and potential biomarkers. In addition to mediating many of the regenerative effects attributed to MSCs, EVs contain proteins, messenger RNAs, lipids, and regulatory miRNAs that reflect the biological status of both transplanted cells and host tissues. Consequently, circulating EVs represent a promising minimally invasive “liquid biopsy” capable of providing real-time information on regenerative activity, inflammatory status, and treatment response without the need for repeated tissue sampling [27,28]. Beyond miRNAs and EVs, circulating inflammatory cytokines (e.g., IL-1β, TNF-α), matrix degradation products (e.g., CTX-II and COMP), chemokines, and other protein-based biomarkers may further improve the biological characterisation of IVD degeneration and complement conventional imaging findings. The integration of quantitative MRI biomarkers with molecular profiling has the potential to improve patient stratification, enable objective monitoring of regenerative responses, and support personalised therapeutic strategies by identifying patients most likely to respond to MSC-based therapies. Such multimodal biomarker approaches represent an important step towards precision regenerative medicine for IVD degeneration. Although most molecular biomarkers remain in the preclinical or early translational stage, their integration with advanced imaging and standardised clinical outcome measures is expected to support patient selection, facilitate objective monitoring of biological regeneration, and accelerate the clinical translation of MSC-based therapies for intervertebral disc degeneration [28,29,30].

### 5.4. Biomarkers for Predicting Therapeutic Response

One of the major challenges in regenerative medicine is the marked variability in clinical outcomes following MSC therapy. Biological heterogeneity between patients, differences in disease stage, and variations in the inflammatory microenvironment likely contribute to inconsistent treatment responses [31,32,33]. Future studies should therefore focus on developing multimodal biomarker panels that combine quantitative MRI parameters, inflammatory cytokines, extracellular matrix degradation products, and molecular biomarkers to predict therapeutic efficacy before treatment initiation [33,34]. Such integrated diagnostic approaches could substantially improve patient selection, optimise treatment timing, enable objective monitoring of tissue regeneration, and facilitate the standardisation of future clinical trials. As regenerative medicine increasingly moves towards precision medicine, validated diagnostic biomarkers will become indispensable for translating MSC-based therapies into routine clinical practice [1,2,35]. Ultimately, the integration of quantitative imaging, molecular profiling, and artificial intelligence-driven biomarker analysis may enable precision regenerative medicine by supporting individualised patient selection, predicting treatment response, and objectively monitoring the biological efficacy of MSC-based therapies [35,36,37].

## 6. MSC-Based Regenerative Mechanisms

**Figure 2:**
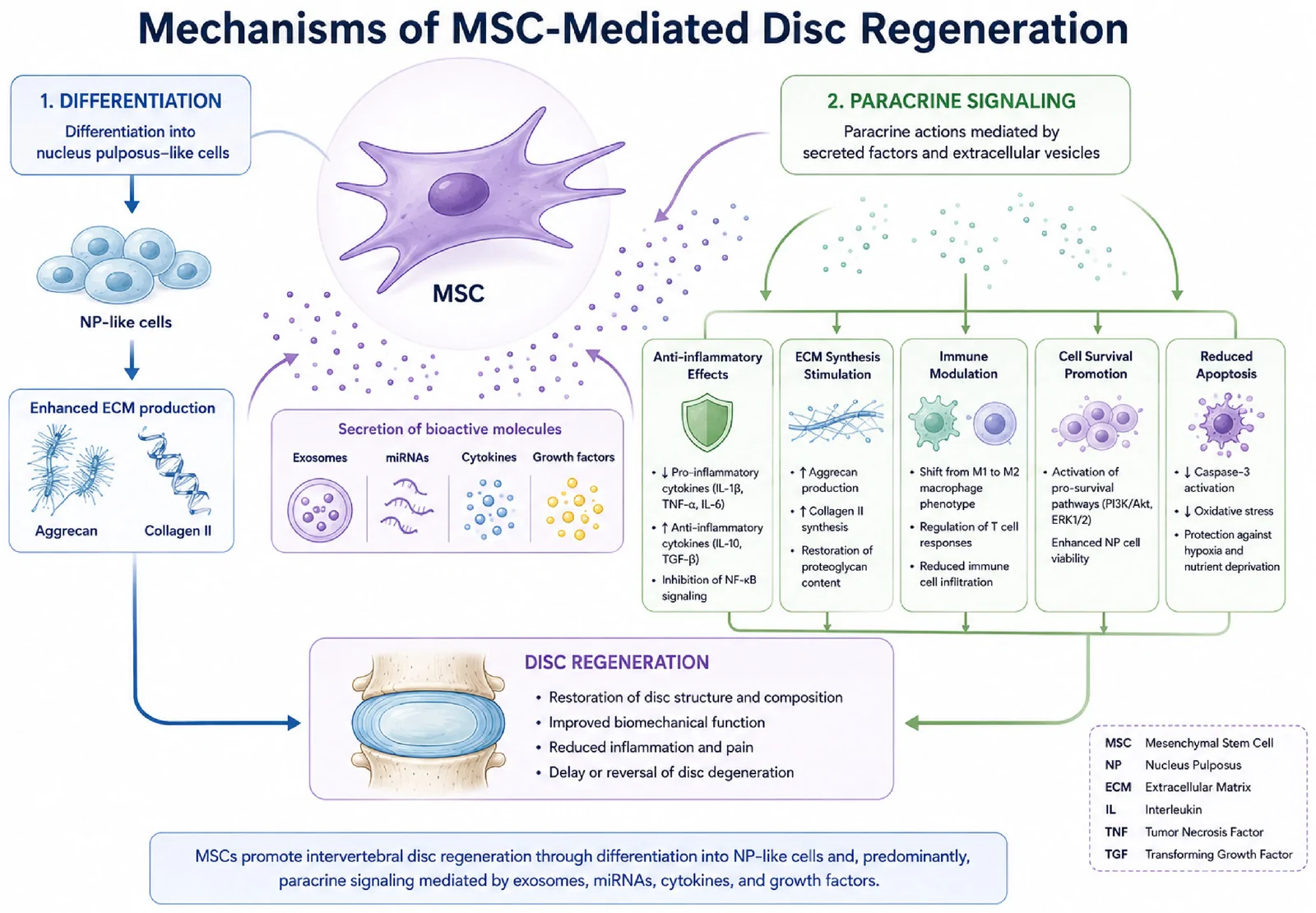
Mechanisms of MSC-mediated intervertebral disc regeneration. MSCs may contribute to intervertebral disc regeneration through direct differentiation and paracrine signalling. Although MSCs can acquire nucleus pulposus (NP)-like characteristics and promote extracellular matrix (ECM) production under appropriate experimental conditions, their long-term survival and functional integration within the hostile microenvironment of the degenerated disc are limited. Current evidence therefore suggests that MSC-mediated therapeutic effects are predominantly driven by paracrine signalling through secreted bioactive factors and extracellular vesicles, including exosomes, regulatory miRNAs, cytokines, and growth factors. These mediators exert anti-inflammatory and immunomodulatory effects, stimulate ECM synthesis, promote NP cell survival, and reduce apoptosis, thereby supporting restoration of disc homeostasis and potentially slowing the progression of intervertebral disc degeneration.

MSCs may promote intervertebral disc regeneration through several complementary mechanisms [38,39]. Early research emphasised their potential to differentiate into nucleus pulposus-like cells and directly contribute to tissue repair. However, limited long-term survival and engraftment within the hostile disc microenvironment suggest that direct cellular replacement plays a relatively limited role in vivo. Increasing evidence instead indicates that the therapeutic effects of MSCs are mediated predominantly through paracrine signalling, including the secretion of bioactive molecules and extracellular vesicles [40,41]. The following sections summarize these interconnected mechanisms, with particular emphasis on secretome- and extracellular vesicle-mediated effects. The principal mechanisms of MSC-mediated intervertebral disc regeneration are summarised in Figure 2.

### 6.1. MSC Differentiation

Early studies of MSC-based intervertebral disc regeneration focused on the capacity of transplanted MSCs to differentiate into nucleus pulposus (NP)-like cells and directly contribute to tissue repair. Under appropriate experimental conditions, MSCs derived from bone marrow, adipose tissue, and umbilical cord can acquire NP-like phenotypic characteristics, including increased expression of aggrecan, collagen type II, SOX9, and other disc-associated markers [1,2,40,41]. Growth factors such as transforming growth factor-β (TGF-β), growth differentiation factor-6 (GDF-6), and bone morphogenetic proteins (BMPs), together with hypoxic culture conditions and biomaterial-based scaffolds, can promote discogenic differentiation and extracellular matrix production [40,41].

However, the contribution of direct MSC differentiation to regeneration in vivo appears to be limited by the hostile microenvironment of the degenerated disc, which is characterised by severe hypoxia, nutrient deprivation, acidic pH, inflammation, and mechanical stress. These conditions restrict the survival, persistence, and functional integration of transplanted cells, and only a limited proportion of administered MSCs are expected to persist long term [3]. Consequently, although differentiation into NP-like cells may contribute to regenerative effects under favourable conditions, current evidence suggests that it is unlikely to represent the predominant mechanism of MSC-mediated disc repair. Instead, the therapeutic effects of MSCs are increasingly attributed to paracrine signalling and secretome-mediated mechanisms, including extracellular vesicle-mediated intercellular communication [3,39].

### 6.2. Paracrine Signalling

MSCs can influence the degenerated disc through the secretion of biologically active mediators that act on resident disc and immune cells and stimulate endogenous repair mechanisms [1,6]. These paracrine signals suppress inflammatory mediators such as interleukin-1β (IL-1β) and tumour necrosis factor-α (TNF-α), reduce NP cell apoptosis, and promote anabolic ECM synthesis. They can also attenuate matrix degradation by suppressing matrix metalloproteinases (MMPs) while enhancing aggrecan and collagen type II production [2,7]. In addition, MSC-mediated immunomodulation can modify the inflammatory microenvironment of the degenerated disc. Together, these effects provide a mechanistic explanation for regenerative responses despite limited long-term MSC survival after transplantation [1,2,3,7].

### 6.3. MSC Secretome

The bioactive products released by MSCs are collectively referred to as the MSC secretome and include soluble proteins, growth factors, cytokines, chemokines, lipids, messenger RNAs, microRNAs, and EVs [1,42]. Through these components, the secretome regulates processes relevant to disc regeneration, including inflammation, apoptosis, oxidative stress, ECM turnover, angiogenesis, and cellular metabolism [2,6,43,44].

Recognition of the secretome as an important mediator of MSC activity has shifted attention from direct cellular replacement towards the signalling functions of MSCs and has provided the rationale for developing cell-free regenerative approaches. Among secretome components, EVs, particularly exosomes, have received considerable attention as potential therapeutic mediators [43,44].

### 6.4. Extracellular Vesicles and Exosomes

EVs are membrane-bound particles released by cells and are commonly classified into exosomes, microvesicles, and apoptotic bodies according to their size and biogenesis [8,9]. They mediate intercellular communication by transferring proteins, lipids, mRNAs, miRNAs, and other regulatory molecules to recipient cells.

MSC-derived exosomes have demonstrated anti-inflammatory, anti-apoptotic, and matrix-supportive effects in experimental models of IVD degeneration [2,8]. Their activity is partly mediated by regulatory miRNAs, including miR-21, miR-26a, miR-31-5p, miR-142-3p, miR-223, and miR-532-5p, which have been implicated in the regulation of inflammation, oxidative stress, autophagy, mitochondrial function, ECM metabolism, and cell survival [8]. These effects involve signalling pathways such as NF-κB, PI3K/Akt, MAPK, and Wnt/β-catenin. The ability of EVs to reproduce important paracrine effects of MSCs without requiring transplantation of viable cells has provided the basis for the development of cell-free regenerative strategies, which are discussed in the following section [2,3,4,8].

## 7. Cell-Free Therapy

The growing recognition that many of the regenerative effects of MSCs are mediated by secreted factors, particularly EVs, has stimulated interest in cell-free approaches for intervertebral disc regeneration. Among these, MSC-derived exosomes have received particular attention as potential therapeutic agents that may reproduce key paracrine effects of MSCs without requiring transplantation of viable cells [1,2,3].

### 7.1. Advantages of Cell-Free Therapy

Compared with conventional MSC therapy, exosome-based approaches offer several potential safety and practical advantages. As cell-free biological products, exosomes do not possess the proliferative or differentiation capacity of viable MSCs and may therefore reduce concerns related to ectopic tissue formation and tumourigenicity associated with cell-based therapies. However, their long-term safety following intradiscal administration remains to be established [2,3]. Their generally reported low immunogenicity may also support the development of allogeneic, ‘off-the-shelf’ therapeutic products, although immune responses may depend on exosome composition, source, manufacturing, and administration conditions [4].

From a translational perspective, exosomes may offer greater stability during storage and transportation and improved potential for batch standardisation compared with viable MSCs [6]. Their small size may additionally facilitate distribution through the dense extracellular matrix of the avascular intervertebral disc following intradiscal administration [2]. Together with the regenerative activity demonstrated in experimental models, these characteristics make exosomes attractive candidates for the development of cell-free regenerative strategies for IVD degeneration [4,6,7,8].

### 7.2. Current Limitations

Despite these potential advantages, several challenges currently limit the clinical implementation of exosome-based therapies. A major obstacle is the lack of standardised methods for exosome isolation, purification, and characterisation. Commonly used techniques, including ultracentrifugation, size-exclusion chromatography, ultrafiltration, polymer-based precipitation, and immunoaffinity capture, can yield vesicle preparations with variable purity, recovery, and biological activity, complicating comparisons across studies [3].

Exosome composition is also influenced by characteristics of the parent MSCs and their culture conditions, including tissue source, donor age, passage number, oxygen tension, and inflammatory priming. Consequently, differences in production protocols may result in substantial variability in molecular cargo and therapeutic activity [2,4].

Important questions regarding administration also remain unresolved, including optimal dose, treatment frequency, injection volume, biodistribution, and long-term persistence following intradiscal delivery. Furthermore, validated potency assays and universally accepted quality-control standards have not yet been established, representing important obstacles to regulatory approval and large-scale manufacturing [6]. Unlike viable MSCs, which can respond dynamically to changes in their environment, exosomes contain a predefined molecular cargo and cannot adapt their secretory profile after administration [3,4,6,7]. Addressing these biological, technical, and manufacturing limitations will therefore be essential for the development of reproducible exosome-based therapies.

### 7.3. Clinical Translation

Clinical translation of MSC-derived exosomes for IVD regeneration remains at an early stage. Current evidence is derived predominantly from in vitro experiments and preclinical animal studies, while direct clinical evidence remains limited [2,6]. No exosome-based therapy has yet been approved specifically for the treatment of IVD degeneration. Progress towards clinical application will require reproducible GMP-compliant manufacturing, validated potency and quality-control assays, defined dosing and administration strategies, and robust long-term safety and efficacy data. Harmonised regulatory frameworks and well-designed randomised clinical trials will also be necessary to establish whether the promising effects observed in experimental models translate into clinically meaningful benefits and to determine the role of exosome-based approaches relative to conventional MSC therapy [2,3,4,6,44].

### 7.4. MSC Therapy Versus Exosome-Based Therapy

Conventional MSC therapy and MSC-derived exosome therapy share several regenerative mechanisms but differ in important biological and translational characteristics [45,46]. Living MSCs remain metabolically active after implantation and can respond dynamically to the host microenvironment through continuous secretion of trophic factors and EVs. Under appropriate conditions, they may also differentiate towards nucleus pulposus-like cells and contribute directly to tissue repair [46,47]. However, their therapeutic activity may be limited by poor survival within the hostile environment of the degenerated disc, as well as donor variability, cellular senescence, manufacturing complexity, and potential cell leakage following intradiscal administration [7]. In contrast, exosome-based approaches are not dependent on the survival of transplanted cells and may offer advantages in terms of immunogenicity, storage, and product standardisation. However, their activity is determined by the molecular cargo present at the time of administration, and clinical evidence supporting their efficacy in IVD regeneration remains limited [48,49]. Thus, the two approaches should be regarded as potentially complementary rather than mutually exclusive regenerative strategies. Future strategies may combine the advantages of both approaches through biomaterial-assisted delivery, engineered exosomes enriched with therapeutic microRNAs, or systems combining living MSCs with controlled exosome release. Such approaches may enhance local retention and regenerative activity while addressing some of the limitations associated with either strategy alone [49,50,51]. The principal differences between conventional MSC therapy and MSC-derived exosome therapy are summarised in Table 1.

## 8. Comparison of Mesenchymal Stromal Cell Sources

**Figure 3:**
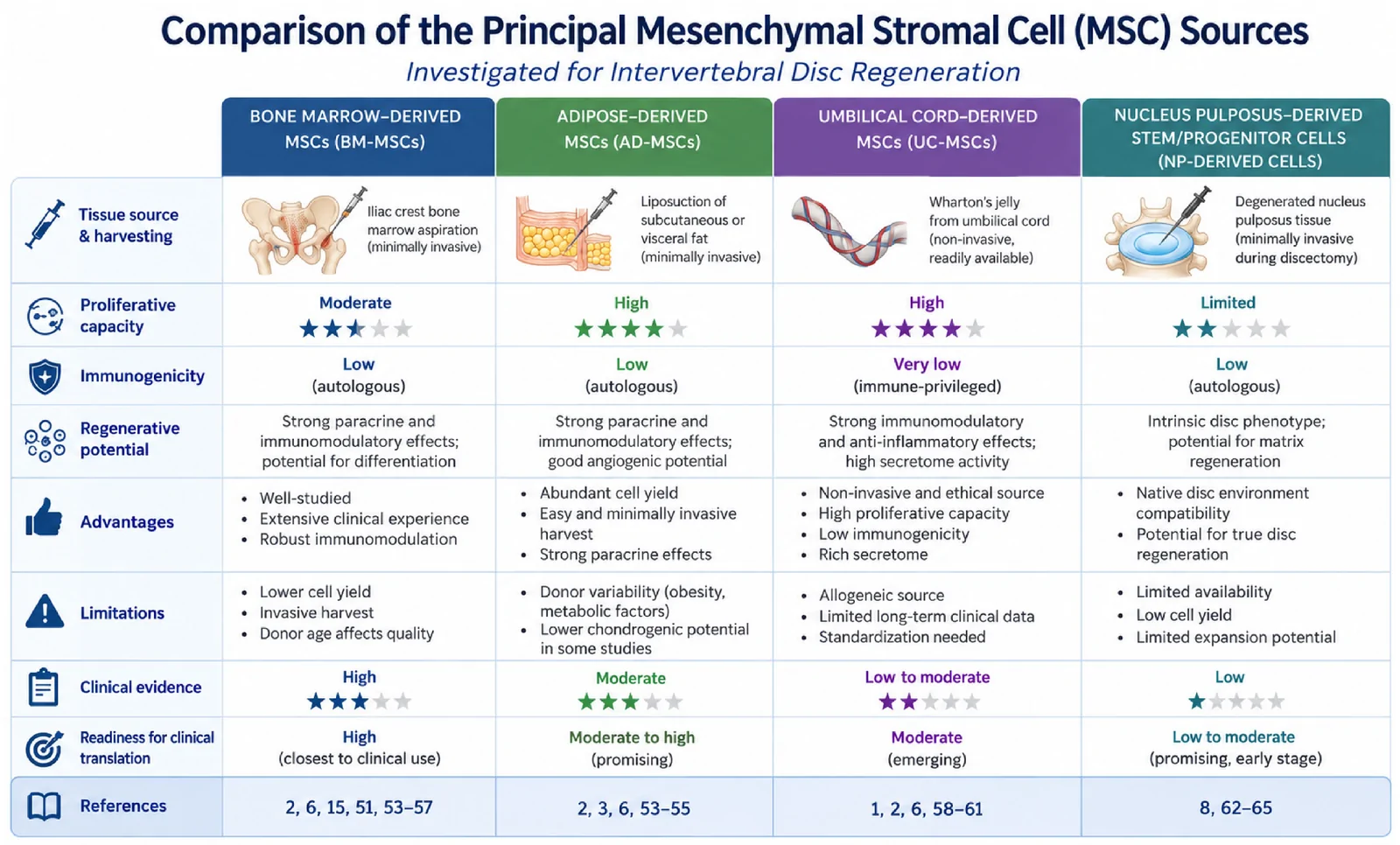
Comparison of the principal MSC sources investigated for intervertebral disc regeneration. Bone marrow-derived MSCs (BM-MSCs), adipose-derived MSCs (AD-MSCs), umbilical cord-derived MSCs (UC-MSCs), and nucleus pulposus-derived stem/progenitor cells (NP-derived cells) differ in their biological properties and translational potential. The figure summarises the main advantages and limitations of each cell source, together with their harvesting methods, proliferative capacity, immunogenicity, level of clinical evidence, and overall readiness for clinical translation. Although no single MSC source has demonstrated clear superiority, each offers distinct advantages that may be suited to specific regenerative strategies and clinical applications [1,2,3,6,8,51,53,54,55,56,57,58,59,60,61,62,63,64,65].

The choice of MSC source is a critical determinant of the success of regenerative therapies for IVD degeneration. Although MSCs derived from bone marrow, adipose tissue, umbilical cord, and nucleus pulposus exhibit similar multilineage differentiation capacity and immunomodulatory properties, they differ considerably in proliferation potential, ease of harvesting, biological behaviour, and level of clinical evidence. Consequently, selecting the most appropriate cell source requires balancing biological performance with translational feasibility rather than identifying a universally superior MSC population [1,2]. The principal characteristics of the most commonly investigated MSC sources for intervertebral disc regeneration are summarised in Figure 3.

### 8.1. Bone Marrow-Derived MSCs

Bone marrow-derived MSCs (BM-MSCs) remain the most extensively investigated cell population for IVDD regeneration and currently provide the strongest preclinical and clinical evidence. BM-MSCs possess robust osteogenic, chondrogenic, and adipogenic differentiation potential together with pronounced immunomodulatory and paracrine activity. Under appropriate biochemical stimulation, they can differentiate toward an NP-like phenotype, increase ECM synthesis, suppress inflammatory signalling, and improve disc hydration in experimental models [51,52].

The principal advantage of BM-MSCs is the extensive body of experimental and clinical data supporting their therapeutic potential. Most published clinical trials evaluating intradiscal MSC therapy have employed BM-MSCs, making them the current benchmark against which alternative cell sources are compared [1,53]. However, bone marrow aspiration is an invasive procedure associated with donor-site morbidity, and both MSC number and regenerative capacity decline with donor age. Since IVD degeneration predominantly affects middle-aged and elderly individuals, age-related cellular senescence may substantially reduce the efficacy of autologous BM-MSC therapy [2,6,53,54,55].

These considerations may be particularly relevant in elderly patients. In addition to age-related changes in the disc microenvironment, ageing may reduce the proliferative and regenerative properties of autologous MSCs, potentially limiting the effectiveness of autologous cell-based approaches. Allogeneic MSCs from younger donors or cell-free approaches based on MSC-derived extracellular vesicles may offer potential alternatives by reducing dependence on the regenerative capacity of the patient’s own cells. However, clinical evidence specifically evaluating these strategies in elderly patients remains limited, and advanced structural degeneration may itself restrict the capacity for biological repair. Therefore, chronological age alone should not be regarded as a definitive criterion for treatment suitability; future studies should determine how age, biological characteristics of the disc, degeneration stage, and MSC source interact to influence therapeutic response [15,56,57].

### 8.2. Adipose-Derived MSCs

Adipose-derived MSCs (AD-MSCs) have emerged as an attractive alternative because adipose tissue is abundant, harvesting by liposuction is minimally invasive, and significantly larger numbers of stromal cells can be obtained than from bone marrow. AD-MSCs also exhibit rapid proliferation and high expansion capacity, facilitating large-scale manufacturing for regenerative applications [2,6,54,55]. Experimental evidence indicates that AD-MSCs possess discogenic potential comparable to BM-MSCs. In particular, stimulation with growth differentiation factor-6 (GDF-6) significantly enhances differentiation toward an NP-like phenotype, increases aggrecan production, and promotes the formation of a proteoglycan-rich extracellular matrix. Interestingly, several studies suggest that AD-MSCs may respond more strongly to GDF-6 stimulation than BM-MSCs, potentially making them particularly suitable for disc regeneration strategies based on growth factor induction [2,3]. Despite these advantages, several uncertainties remain. Adipose tissue naturally favours adipogenic differentiation, raising questions regarding the intrinsic discogenic potential of AD-MSCs. Furthermore, although preclinical results are highly encouraging, clinical experience with AD-MSCs remains considerably more limited than with BM-MSCs, and additional randomised clinical trials are required to establish their long-term efficacy and safety [2,53,54].

### 8.3. Umbilical Cord-Derived MSCs

Umbilical cord-derived MSCs (UC-MSCs) have attracted increasing attention because they combine high proliferative capacity with low immunogenicity and potent immunomodulatory activity [58,59]. As neonatal cells, UC-MSCs exhibit reduced cellular senescence, longer telomeres, and greater proliferative potential than adult tissue-derived MSCs. Their collection is non-invasive and raises few ethical concerns because umbilical cords are normally discarded after delivery [1,2,60]. These characteristics make UC-MSCs particularly attractive for standardised allogeneic “off-the-shelf” therapies. Experimental studies have demonstrated that UC-MSCs suppress inflammatory cytokine production, promote ECM synthesis, and improve disc morphology in animal models of IVD degeneration. Moreover, UC-MSC-derived extracellular vesicles and exosomes have shown promising regenerative effects by modulating apoptosis, oxidative stress, mitochondrial function, and inflammation [2,6]. Nevertheless, despite their favourable biological properties, clinical evidence supporting UC-MSC therapy remains limited. Long-term safety, optimal dosing, and comparative efficacy relative to BM-MSCs have yet to be established through adequately powered randomised clinical trials [2,61].

### 8.4. Nucleus Pulposus-Derived Stem/Progenitor Cells

Nucleus pulposus-derived stem/progenitor cells (NP-MSCs or NPPCs) represent a unique regenerative cell population because they originate directly from the native disc microenvironment [62,63]. Consequently, these cells are naturally adapted to hypoxia, hyperosmolarity, acidic pH, and limited nutrient availability, conditions that frequently impair the survival of exogenously transplanted MSCs. NP-derived progenitor cells also express NP-associated markers and synthesize extracellular matrix components characteristic of healthy disc tissue, suggesting superior biological compatibility with the intervertebral disc [8].

However, several important translational barriers currently limit their clinical applicability. NP-derived stromal cells require surgical harvesting, only limited numbers can be isolated, and cells obtained from degenerated discs frequently exhibit reduced proliferative capacity, impaired differentiation potential, and features of cellular senescence. Consequently, although NP-derived progenitor cells may represent the biologically most appropriate cell source for disc regeneration, their routine clinical application remains technically challenging [8,64,65].

### 8.5. Comparative Perspective

Each MSC source therefore presents a distinct balance of biological advantages and translational limitations. BM-MSCs are supported by the most extensive clinical evidence to date, although their application is constrained by invasive harvesting procedures and age-related declines in cellular regenerative capacity. AD-MSCs represent an accessible and abundant cell source with high isolation yields, but their therapeutic efficacy requires further clinical validation. UC-MSCs exhibit considerable proliferative potential and relatively low immunogenicity, making them promising candidates for the development of allogeneic cell-based therapies. In contrast, nucleus pulposus-derived progenitor cells possess characteristics that may favour their adaptation to the intervertebral disc microenvironment, although their clinical application remains limited by challenges associated with tissue harvesting and the restricted availability of suitable cells [66,67,68].

Taken together, the available evidence indicates that no single MSC source can be considered universally superior for IVD regeneration. Rather, the selection of the most appropriate cell population should be guided by the specific therapeutic objectives, individual patient characteristics, and the delivery strategy employed [67,68]. Future comparative studies performed under standardised experimental and clinical conditions will be essential to determine which MSC source provides the greatest regenerative efficacy while maintaining safety, reproducibility, and scalability. Furthermore, advances in biomaterials, genetic engineering, hypoxic preconditioning, and extracellular vesicle-based therapies may ultimately prove more influential than the choice of cell source alone in determining clinical outcomes [1,2,68,69,70].

## 9. Intervertebral Disc Regeneration: Evidence from Preclinical Animal Models

Although nucleus pulposus cells constitute only about 1% of the total intervertebral disc volume, they are essential for maintaining the ECM, which underpins disc architecture, hydration, and biomechanical performance [10]. These cells regulate the synthesis and turnover of key matrix components, including proteoglycans and type II collagen, enabling the disc to retain water and resist compressive forces. Their loss or dysfunction during degeneration therefore represents a major target for regenerative therapy [10,18].

One of the first attempts to restore disc cellularity was described by Nishimura and Mochida using a rat model [71]. In that landmark experiment, cryopreserved autologous nucleus pulposus cells were reimplanted into degenerated discs. The investigators found that this reimplantation delayed annulus fibrosus degeneration and preserved the residual nucleus pulposus compared with untreated controls. This study provided the earliest proof of concept that restoring disc cellularity could slow the degenerative process. Subsequent animal studies, ex vivo experiments, and early human investigations further demonstrated that reimplantation of autologous nucleus pulposus cells may reduce degenerative changes, relieve low back pain, and partially restore disc height and hydration compared with discectomy alone [2,18,19,71,72].

Following these early findings, regenerative approaches increasingly focused on multipotent stromal cells as a means of replenishing the degenerating disc microenvironment. Among the stromal cell types studied, MSCs have emerged as the most promising for intervertebral disc regeneration. These cells can be delivered into the disc percutaneously, where they may support matrix repair, modulate inflammation, and promote the formation of disc-like tissue. MSCs are able to differentiate into multiple mesenchymal lineages and, under suitable conditions, may acquire phenotypic traits resembling nucleus pulposus cells [6].

Both BM-MSCs and adipose-derived MSCs (AD-MSCs) have demonstrated the ability to differentiate into nucleus pulposus-like cells in vitro. Beyond differentiation, MSCs exhibit pronounced trophic and immunomodulatory actions through paracrine signalling. By releasing anabolic growth factors and cytokines, they can influence the behaviour of resident nucleus pulposus cells, stimulate ECM production, and suppress inflammatory cascades involved in disc degeneration. These features make MSCs particularly attractive for regenerative strategies in the hostile microenvironment of the degenerated disc [14,20,21,71,72]. Bone marrow remains the most commonly used MSC source in experimental work. In vitro, BM-MSCs can differentiate into osteoblasts, adipocytes, and chondrocytes, identifiable by specific surface marker profiles. Importantly, under appropriate induction conditions, they can also develop phenotypic properties similar to those of native nucleus pulposus cells [1,73]. Some studies have shown that stimulation of BM-MSCs with growth differentiation factor-6 (GDF6) markedly upregulates nucleus pulposus-specific genes, promoting differentiation towards a discogenic phenotype [72].

Numerous in vivo animal studies have provided strong support for the regenerative potential of MSC therapy. Preclinical studies in rat and rabbit models of intervertebral disc degeneration have demonstrated promising regenerative effects following MSC transplantation, including increased disc height and cellularity. Implanted MSCs have been shown to proliferate within the disc and acquire nucleus pulposus-like characteristics, as indicated by the expression of nucleus pulposus-associated markers. Furthermore, these cells contribute to extracellular matrix synthesis, particularly the production of type II collagen and proteoglycans, leading to improvements in disc structure and hydration, as demonstrated by imaging and histological analyses [72,73,74,75,76]. In addition to their differentiation capacity, MSCs exert substantial anti-inflammatory and paracrine effects that may help restore a regenerative environment. Several studies have reported that MSCs secrete bioactive molecules that inhibit pro-inflammatory cytokines such as IL-6, IL-8, and TNF-α, while simultaneously enhancing anabolic pathways involved in matrix synthesis. Such immunomodulatory effects may help neutralise the chronic inflammatory state of degenerated discs and thereby support tissue repair [72,73,74,75]. Further evidence for the therapeutic promise of MSCs comes from large-animal studies with spinal biomechanics closer to those of humans. Experiments with MSC injections in degenerated canine intervertebral discs showed that although marked radiological changes were not detected, treated animals exhibited improved mobility and reduced pain-related behaviour [74,75]. These results suggest that MSC therapy may provide clinically meaningful benefits even when obvious structural regeneration is not apparent, underscoring the importance of assessing both functional and biological endpoints in regenerative studies [76]. More recently, research has focused on improving the regenerative performance of stromal cell therapies [74,75]. Approaches such as hypoxic preconditioning of MSCs, genetic modification to promote discogenic differentiation, and incorporation of hydrogels or biomaterial scaffolds have been explored to enhance cell survival and retention within the disc. These strategies aim to counteract the hostile environment of the degenerated intervertebral disc, which is characterised by low oxygen tension, poor nutrient availability, acidic pH, and high mechanical stress. By providing structural support and biochemical guidance, biomaterial-based delivery systems may further enhance the regenerative effects of transplanted cells [76]. In vitro and in vivo studies consistently support the potential of MSC-based therapies to regenerate degenerated intervertebral discs. Reported effects include restoration of extracellular matrix composition, increased disc height, enhanced hydration as indicated by stronger T2-weighted MRI signals, and suppression of inflammatory mediators. These findings from Crevensten, Sakai, and Miguélez-Rivera support the view that MSCs can promote both structural and functional recovery in degenerated discs [74,75,77]. However, it is important to recognise that most of these data come from preclinical or ex vivo models. Although such studies provide valuable insights into the biology of disc regeneration, their findings cannot be directly translated into clinical practice. Differences in spinal biomechanics, disc dimensions, immune responses, and disease chronicity between animal models and humans may significantly influence therapeutic outcomes. Therefore, carefully designed translational and clinical studies are required to confirm the safety, efficacy, and long-term durability of MSC-based therapies for degenerative disc disease [77,78,79,80,81]. A summary of representative preclinical animal studies investigating cell-based regenerative approaches for the intervertebral disc is presented in Table 2.

## 10. Human Clinical Studies of Intervertebral Disc Regeneration

Although preclinical models have shown promising regenerative effects of MSC therapy in intervertebral disc degeneration, clinical translation remains under active investigation. Over the past two decades, several early-stage clinical studies have assessed the feasibility, safety, and therapeutic potential of MSC-based interventions for degenerative disc disease. While preliminary findings have generally been encouraging, important uncertainties remain regarding the optimal cell source, dose, delivery technique, and durability of benefit. For this reason, MSC therapy has not yet become a standard treatment in routine clinical practice for degenerative disc disease [20,77,78].

One of the earliest clinical reports was published by Yoshikawa et al., who treated two patients with autologous BM-MSCs [82]. Both patients experienced marked pain reduction and improvement in T2-weighted MRI signal intensity in the treated discs, suggesting better hydration and possible regenerative activity. Although the study was severely limited by the very small number of patients, it provided valuable initial clinical support for the feasibility of MSC-based disc treatment. Subsequent studies built on these early observations. Orozco et al. treated ten patients with chronic discogenic low back pain using intradiscal injections of autologous MSCs [55]. Although MRI did not show a statistically significant increase in disc height, better disc hydration was observed, and nearly 85% of patients reported considerable pain relief within three months of treatment [55,83]. These clinical improvements were also associated with better functional performance and quality of life. Similarly, Pettine et al. assessed autologous BM-MSC injections in 26 patients and found statistically significant pain reduction in 21, along with improvement in disability scores and functional outcomes [84]. Further observational reports have also supported the therapeutic potential of MSC treatment. Centeno et al. described improvements in both symptoms and radiological appearance in 33 patients who received autologous MSC injections [85]. Their study showed reductions in disc protrusion and improvement in disc morphology on imaging, suggesting that structural changes may accompany symptom relief. Additional progress was reported by Elabd et al., who used BM-MSCs preconditioned under hypoxic culture conditions before implantation [86]. Hypoxic preconditioning is thought to improve MSC survival and activity within the low-oxygen disc environment. In that study, five patients received intradiscal injections of these conditioned cells, and all reported clinical improvement, while four of the five also experienced greater muscle strength and mobility. These observations highlight the possible importance of refining cell preparation methods to improve therapeutic performance. Despite these favourable findings, several methodological limitations restrict the interpretation and generalisability of the early clinical literature. Most studies involved small cohorts and lacked robust control groups or randomised designs. In addition, differences in patient selection, cell source, culture protocols, and injection technique make direct comparisons between studies difficult. Because of these limitations, it remains uncertain to what extent the observed improvements reflect the true effects of stromal cell therapy rather than placebo responses, natural fluctuations in symptoms, or concurrent conservative treatment. Here, it is important to distinguish bone marrow concentrate (BMC) from culture-expanded BM-MSCs. BMC is a heterogeneous preparation obtained by concentrating bone marrow aspirate and contains multiple cell populations, with mesenchymal stromal/progenitor cells representing only a small fraction. In contrast, culture-expanded BM-MSCs undergo ex vivo isolation and expansion to enrich the mesenchymal stromal cell population before administration. Consequently, the total nucleated cell dose reported for BMC cannot be directly compared with the MSC dose used in studies employing culture-expanded BM-MSCs, and this distinction should be considered when interpreting clinical outcomes across studies [79,80,81].

In response to these limitations, more recent investigations have attempted to adopt stronger study designs. A randomised controlled trial reported by Amirdelfan et al., which enrolled 100 patients with chronic discogenic low back pain, demonstrated statistically significant reductions in pain and improvements in functional outcomes in patients treated with MSCs compared with controls [87]. The treatment also showed a favourable safety profile, with no major adverse events reported. Nevertheless, even this larger study highlighted persistent challenges, including variation in cell preparation and delivery procedures, possible selection bias, and the need for longer-term follow-up to determine the persistence of benefit.

Another important limitation of current clinical research concerns outcome assessment. Many studies rely mainly on patient-reported outcomes such as pain scales and disability questionnaires. Although these are essential indicators of clinical benefit, they remain subjective and can be influenced by psychological and contextual factors. Therefore, objective measures such as quantitative MRI, T2 mapping, and biochemical markers of disc metabolism are increasingly recognised as valuable complementary tools for evaluating structural repair and biological regeneration within the disc [84,85].

When interpreting these clinical studies, it is important to distinguish symptomatic and functional outcomes from structural indicators of disc regeneration. Clinical outcomes, including reductions in pain and disability and improvements in function and quality of life, should be considered separately from structural changes assessed by imaging, such as disc hydration, T2-weighted MRI signal, disc height, or Pfirrmann grade. Improvements in these clinical parameters may occur in the absence of measurable structural regeneration and therefore should not alone be interpreted as evidence of successful disc regeneration [55,84,87].

Overall, the currently available clinical evidence suggests that MSC-based therapies may provide meaningful improvements in pain and functional status for patients with degenerative disc disease. However, the data remain preliminary, and the mechanisms responsible for these benefits are still not fully understood. It remains uncertain whether the observed effects arise mainly from true structural regeneration, suppression of inflammation, or paracrine signalling [78,84,85].

Importantly, clinical improvement should not be considered synonymous with structural or biological regeneration of the intervertebral disc. Reductions in pain and disability, reflected by outcomes such as VAS and ODI, represent clinically meaningful symptomatic and functional benefits but do not directly demonstrate restoration of disc tissue [22,23,24]. Evidence of regeneration requires complementary structural or biological endpoints, such as changes in disc hydration, composition, height, quantitative MRI parameters, or other validated biomarkers of tissue recovery. Accordingly, clinical and regenerative outcomes should be interpreted separately when evaluating the efficacy of MSC-based therapies. Although several studies have reported improvements in pain and function, evidence for consistent structural regeneration of the intervertebral disc remains considerably more limited [77,78,79].

To establish MSC therapy as a dependable clinical treatment for degenerative disc disease, further large, multicentre randomised controlled trials will be necessary. Future studies should work towards standardisation of cell processing, refinement of dosing and delivery protocols, and inclusion of objective imaging and molecular biomarkers to assess therapeutic response. Long-term follow-up will also be critical to determine both durability of benefit and long-term safety. Only through rigorous and standardised clinical evaluation will it be possible to define the true therapeutic value of MSC therapy for degenerative disc disease [78,79]. A summary of the clinical studies investigating MSC therapy for degenerative disc disease in humans is presented in Table 3.

## 11. Translational, Biological and Technical Challenges

**Figure 4:**
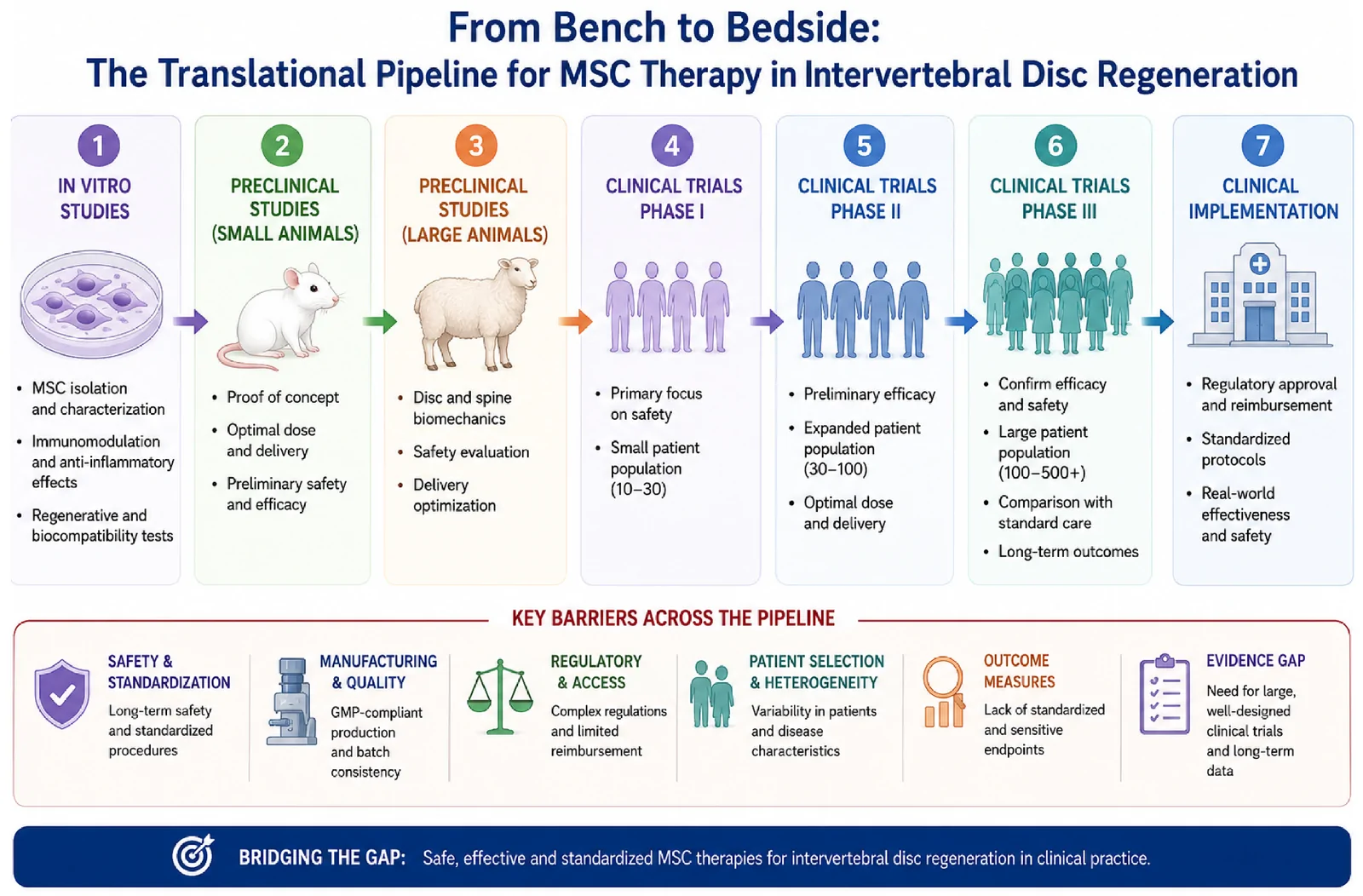
Experimental-to-clinical translation pipeline of MSC-based therapy for intervertebral disc regeneration. MSC therapy progresses from in vitro studies and preclinical animal models to clinical trials and, ultimately, routine clinical implementation. Despite encouraging experimental results, clinical translation remains limited by biological, technical, and regulatory barriers, including poor cell survival, manufacturing variability, heterogeneous clinical protocols, regulatory requirements, and the lack of standardised biomarkers and outcome measures. Overcoming these challenges through biomaterial-assisted delivery, cell-free therapies, precision diagnostics, GMP-compliant manufacturing, and well-designed multicentre clinical trials will be essential for translating MSC-based therapies into safe, effective, and standardised treatments for intervertebral disc degeneration.

MSC-based therapies have shown promising regenerative potential in preclinical models of IVD degeneration, with reported benefits including increased ECM synthesis, preservation of disc height, improved tissue hydration, and reduced inflammatory activity [1,2]. Nevertheless, the translation of these experimental findings into clinical practice remains challenging. Although early clinical investigations have reported improvements in pain and functional outcomes, robust evidence supporting sustained structural regeneration is still lacking. Furthermore, substantial variability in patient selection criteria, MSC sources, cell manufacturing procedures, therapeutic doses, administration techniques, and outcome measures complicates the interpretation and comparison of clinical findings. These challenges indicate that the gap between experimental success and clinical implementation arises not from a single obstacle but from the complex interplay of biological, biomechanical, technical, and methodological limitations [3,65,79]. To illustrate the central concept of this review, Figure 4 presents the experimental-to-clinical translation pathway of MSC-based therapy and highlights the major biological, technical, methodological, and regulatory challenges that must be overcome to bridge the gap between promising preclinical findings and routine clinical application.

The major translational barriers limiting the clinical implementation of MSC-based therapy for IVD degeneration and emerging strategies to overcome them are summarised in Table 4.

### 11.1. Biomaterial-Assisted Delivery Strategies: Current Platforms and Translational Challenges

Biomaterial-assisted delivery systems have emerged as promising strategies to overcome several limitations associated with direct intradiscal administration of MSCs, particularly poor cell retention, limited survival, and insufficient mechanical support within the IVD degeneration. An effective delivery platform must simultaneously provide a favourable microenvironment for transplanted cells, withstand the mechanical demands of the spinal segment, and support ECM regeneration without compromising the structural integrity of the disc. However, achieving an appropriate balance between biological compatibility and biomechanical functionality remains a major challenge [88,89].

Injectable hydrogels represent one of the most extensively investigated biomaterial platforms for IVD regeneration. Natural polymers, including collagen, gelatin, hyaluronic acid, alginate, and chitosan, have attracted considerable interest because of their biocompatibility, high water content, and ability to provide an ECM-like environment [88,89,90]. These properties may support MSC survival, proliferation, and regenerative activity following intradiscal administration. Nevertheless, natural hydrogels frequently exhibit insufficient mechanical strength, limited resistance to repeated loading, and variable degradation characteristics. Their biological composition may also introduce batch-to-batch variability, complicating material standardisation and reproducibility. Consequently, although natural hydrogels offer favourable biological properties, their capacity to provide durable mechanical support within the load-bearing IVD remains uncertain [88,89].

Synthetic polymers, including polyethylene glycol-based hydrogels and other engineered polymeric networks, provide greater control over material composition, crosslinking density, degradation behaviour, and mechanical properties [90]. However, synthetic materials may lack the intrinsic biological signals required for optimal cell adhesion and matrix production. To address these limitations, hybrid and composite hydrogels combining natural and synthetic components have been developed to integrate biological functionality with improved structural stability. Interpenetrating polymer networks and reinforced hydrogel systems are particularly promising because they enable simultaneous modification of mechanical and biological properties. Nevertheless, increasing scaffold stiffness may adversely affect cellular behaviour, highlighting the need to optimise material properties rather than maximising mechanical strength alone [89]. A systematic review of injectable cell-delivery biomaterials for IVD repair identified a recurring trade-off between biomechanical performance and cellular outcomes, emphasising the importance of developing materials that provide adequate initial mechanical support while permitting subsequent cell-mediated tissue regeneration [89,90].

Beyond conventional hydrogels, advanced biomaterial platforms increasingly incorporate bioactive molecules, extracellular vesicles, or other therapeutic agents to improve the regenerative microenvironment. Stimuli-responsive hydrogels designed to react to local biochemical conditions, including inflammatory or oxidative stress-related changes, offer opportunities for more controlled therapeutic release. Decellularised ECM-derived hydrogels have also gained attention because they may provide tissue-specific biochemical cues that are not adequately reproduced by conventional polymeric systems. Nevertheless, these approaches introduce additional challenges, including variability in material composition, incomplete control over degradation and release kinetics, and difficulties in achieving consistent manufacturing quality. Furthermore, the release of bioactive agents must be coordinated with the progression of tissue repair to avoid premature depletion or prolonged exposure beyond the intended therapeutic period [91,92].

Three-dimensional bioprinting and scaffold fabrication technologies represent another important direction in IVD tissue engineering. These approaches allow for spatially controlled deposition of cells and biomaterials and may facilitate the development of constructs that more closely reproduce the distinct structural and functional characteristics of the nucleus pulposus and annulus fibrosus [93]. In particular, combinations of hydrogel-based bioinks with mechanically reinforced polymeric scaffolds may improve the integration of biological and structural functions. However, reproducing the complex anisotropic architecture of the native IVD, maintaining cell viability during fabrication, and achieving adequate mechanical resistance under physiological loading remain substantial challenges. In addition, the implantation of larger engineered constructs may require more invasive procedures than injectable systems, potentially limiting their immediate clinical applicability [93,94].

Despite encouraging preclinical findings, the clinical translation of biomaterial-assisted MSC delivery remains limited by several unresolved challenges. The degenerative IVD is characterised by restricted nutrient availability, acidic conditions, inflammation, and substantial mechanical loading, all of which may compromise the long-term survival and functionality of encapsulated cells [88,89]. Biomaterial degradation must also be carefully coordinated with new ECM formation to prevent premature loss of mechanical support or persistence of material that interferes with tissue remodelling. Furthermore, the risk of material leakage or extrusion through annular defects, potential inflammatory responses, and changes in segmental biomechanics require systematic evaluation before clinical application [88,89,95].

Future technological development should therefore prioritise multifunctional biomaterials that combine mechanical stability, biological compatibility, controlled degradation, and sustained therapeutic activity. Particular attention should be directed towards injectable systems with improved resistance to physiological loading, biomimetic materials capable of supporting cell function under degenerative conditions, and composite scaffolds that reproduce the distinct mechanical requirements of individual IVD compartments [89,90,91]. Equally important are standardised manufacturing procedures, reproducible material characterisation, and comprehensive evaluation in physiologically relevant preclinical models. Long-term investigations assessing both biological regeneration and restoration of mechanical function will be essential to establish whether these advanced delivery systems can provide clinically meaningful and durable benefits. Ultimately, successful clinical translation will depend on the development of biomaterials that function not merely as passive MSC carriers but as integrated therapeutic platforms capable of supporting sustained IVD regeneration [89,91,95].

### 11.2. Strategies to Improve Clinical Translation

Overcoming current translational barriers will require coordinated advances in stromal cell biology, tissue engineering, biomaterials science, and clinical trial methodology. More physiologically relevant preclinical models, including large-animal models, ex vivo disc organ culture systems, and biomechanically active bioreactors, are expected to improve the prediction of clinical outcomes by more accurately reproducing the human disc environment [20,65,96].

At the therapeutic level, strategies such as hypoxic preconditioning, genetic modification, optimised biomaterial-assisted delivery, and extracellular vesicle-based therapies may further improve MSC survival, regenerative activity, and long-term efficacy [83,87,97,98,99,100,101]. Equally important will be the implementation of standardised manufacturing protocols, harmonised delivery techniques, objective imaging biomarkers, and adequately powered multicentre randomised clinical trials with long-term follow-up.

Ultimately, successful translation of MSC therapy from laboratory research to routine clinical practice will depend on an integrated multidisciplinary approach combining stromal cell biology, biomechanics, biomaterials engineering, advanced imaging, and rigorous clinical investigation. Through continued optimisation of both biological and technical aspects of therapy, MSC-based regenerative strategies may progressively evolve into effective disease-modifying treatments for intervertebral disc degeneration [78,80,102,103,104,105].

## 12. Standardisation and Regulatory Considerations in MSC Therapy

Successful clinical translation of MSC-based therapies depends not only on biological efficacy but also on standardised manufacturing, rigorous quality control, and harmonised regulatory frameworks. Although encouraging clinical results have been reported, considerable variability in cell production, characterisation, and administration continues to limit reproducibility and comparisons between studies. Consequently, the implementation of GMP-compliant production and standardised quality assurance systems has become a prerequisite for routine clinical application [106,107].

One of the major challenges in the clinical application of MSC-based therapies is the substantial heterogeneity of MSC products. These cells can be obtained from various tissue sources, including bone marrow, adipose tissue, umbilical cord, dental pulp, synovium, and placenta, each characterised by distinct biological and functional properties. Differences in proliferative capacity, differentiation potential, immunomodulatory activity, and secretome composition contribute to variability in therapeutic performance and complicate the standardisation of MSC-based regenerative approaches [107,108]. In addition, donor characteristics, isolation procedures, culture conditions, serum supplementation, oxygen concentration, passage number, and cryopreservation protocols may all influence MSC phenotype and biological potency, contributing to significant batch-to-batch variability [109]. Harmonisation of manufacturing protocols is therefore essential to ensure reproducible therapeutic products.

Standardisation is equally important for clinical administration. Current studies differ considerably with respect to cell dose, injection volume, needle diameter, delivery technique, and the use of biomaterial carriers. Such methodological heterogeneity complicates comparisons between clinical trials and prevents the establishment of evidence-based treatment protocols. Developing standardised delivery procedures will therefore be crucial for optimising therapeutic efficacy and facilitating multicentre studies [106,107].

From a regulatory perspective, MSC-based therapies occupy a complex category that may be classified as biologics, tissue-engineered products, or advanced therapy medicinal products, depending on the degree of cell manipulation and intended clinical application. Regulatory requirements differ substantially between the United States, the European Union, and Japan, particularly regarding minimal manipulation, homologous use, and post-marketing surveillance. Although these frameworks are designed to ensure patient safety, the lack of international harmonisation increases development costs and slows clinical translation [110,111].

Quality control represents another critical challenge. While the International Society for Cell and Gene Therapy (ISCT) has established minimal criteria for defining MSC identity, these phenotypic markers alone do not adequately predict therapeutic potency. Consequently, GMP-compliant manufacturing requires comprehensive assessment of sterility, viability, identity, purity, and, whenever possible, functional potency before product release [111,112]. However, potency testing remains particularly challenging because MSCs exert therapeutic effects through multiple context-dependent mechanisms, including immunomodulation, trophic support, and paracrine signalling. Existing assays, such as T-cell suppression tests, cytokine profiling, and gene-expression-based surrogate markers, have shown promise but have not yet been universally standardised [112].

Long-term safety monitoring also remains essential. Although current clinical evidence suggests that MSC therapy has a favourable safety profile, continued surveillance is necessary to detect rare adverse events, including ectopic tissue formation, immune sensitisation, fibrosis, and potential tumour-supportive effects. Structured pharmacovigilance programmes, patient registries, standardised adverse-event reporting, and complete traceability from donor material to treated patient will therefore be critical for future clinical implementation [113,114,115].

Finally, manufacturing cost and scalability continue to represent significant barriers to widespread clinical adoption. GMP-compliant MSC production requires specialised facilities, validated clean-room environments, highly trained personnel, and extensive quality-control procedures, all of which substantially increase production costs. Emerging technologies, including automated closed-system bioreactors, xeno-free culture media, and large-scale expansion platforms, may improve manufacturing consistency while reducing cost and increasing scalability [107,113].

International collaboration will be essential to overcome these challenges. Consensus initiatives led by organisations such as the ISCT have already contributed substantially to standardising MSC characterisation and manufacturing. Further harmonisation of regulatory requirements, manufacturing standards, potency assays, and multicentre clinical trial design will be necessary to accelerate clinical translation and improve reproducibility across institutions [112,113]. In parallel, advanced ex vivo intervertebral disc organ culture systems and biomechanically active bioreactor platforms are emerging as valuable translational tools for evaluating standardised MSC-based therapies under physiologically relevant conditions. These models enable controlled regulation of oxygen tension, nutrient supply, pH, and mechanical loading, thereby providing a more predictive bridge between conventional in vitro studies and in vivo clinical application [114,115].

Overall, successful clinical implementation of MSC therapy will require the integration of standardised manufacturing, harmonised regulatory frameworks, validated potency testing, and robust multicentre clinical studies. Such coordinated efforts will be essential to ensure the safety, reproducibility, and long-term effectiveness of regenerative therapies for intervertebral disc degeneration [106,107].

## 13. Forthcoming Perspectives and Developing Therapeutic Strategies: Where Is the Field Going Next?

MSC-based therapies have demonstrated considerable potential for the treatment of IVD degeneration, yet several scientific and clinical challenges must still be overcome before they can become part of routine clinical practice. Future research should move beyond establishing proof of concept and prioritise enhancing therapeutic efficacy, refining patient selection criteria, and establishing standardised regenerative approaches capable of achieving sustained clinical benefits. Advances in mesenchymal stromal cell biology, tissue engineering, biomaterial-based delivery systems, and precision diagnostics may help overcome current translational barriers and facilitate the integration of regenerative therapies into clinical practice for intervertebral disc degeneration [116,117]. The principal emerging strategies, their mechanisms of action, potential advantages, and current stage of development are summarised in Table 5.

One promising direction is the optimisation of MSC function before transplantation. Cell preconditioning using hypoxia, inflammatory mediators, or metabolic stress has emerged as an effective strategy to enhance MSC survival and regenerative activity within the hostile intervertebral disc environment. In particular, hypoxic preconditioning activates adaptive metabolic pathways that improve resistance to low oxygen tension, enhance extracellular matrix production, increase secretion of trophic factors, and reduce apoptosis following implantation. Such approaches may substantially improve the therapeutic performance of MSCs without altering their fundamental biological characteristics [116,117].

Genetic engineering represents another rapidly evolving area of regenerative medicine. Advances in gene-editing technologies, including CRISPR-based platforms, enable targeted modification of MSCs to increase the expression of anabolic growth factors such as growth differentiation factor-6 (GDF-6), transforming growth factor-β (TGF-β), and bone morphogenetic proteins. These engineered cells may exhibit enhanced discogenic differentiation, improved extracellular matrix synthesis, and greater resistance to the degenerative disc microenvironment, potentially increasing the durability of regenerative outcomes [116].

The development of cell-free regenerative therapies also represents an important future direction. As discussed in previous sections, extracellular vesicles and exosomes reproduce many of the beneficial paracrine effects of MSCs while avoiding several limitations associated with living cell transplantation. Experimental studies have demonstrated their anti-inflammatory, anti-apoptotic, and matrix-supportive properties, making them attractive candidates for standardised “off-the-shelf” biological therapies. Future research will need to optimise exosome manufacturing, cargo engineering, targeted delivery, and large-scale production before these approaches can be translated into clinical practice [117,118,119].

Continued advances in tissue engineering are expected to further improve regenerative outcomes. Injectable hydrogels, biomaterial scaffolds, and three-dimensional bioprinted constructs may enhance cell retention, provide mechanical support, and enable controlled release of growth factors or extracellular vesicles within the degenerated disc. Combining MSC therapy with biomaterial-assisted delivery systems may therefore overcome several limitations of conventional intradiscal cell injection while better replicating the native architecture of the intervertebral disc [116,119].

Precision medicine is also likely to play an increasingly important role in future regenerative therapies. IVD degeneration is a biologically heterogeneous disease, and treatment efficacy is likely to depend on patient-specific factors such as disease stage, disc microenvironment, and molecular profile [117]. The integration of molecular biomarkers, advanced quantitative imaging, and machine-learning approaches may facilitate identification of patients most likely to benefit from MSC-based interventions. In particular, patients with early-stage degeneration may represent more suitable candidates than those with advanced disc collapse, in whom the regenerative potential is substantially reduced [120].

Ultimately, successful clinical translation will require large multicentre randomised controlled trials with standardised manufacturing protocols, delivery strategies, imaging endpoints, and long-term follow-up. These investigations will be critical for determining the durability of therapeutic outcomes, establishing long-term safety and efficacy, and developing evidence-based treatment protocols that meet regulatory requirements and facilitate broader clinical adoption [2,120].

In summary, the future of regenerative therapy for IVD degeneration will likely depend on the integration of stromal cell biology, genetic engineering, tissue engineering, biomaterials science, and precision medicine. Through continued technological innovation and rigorous clinical validation, MSC-based therapies have the potential to evolve from promising experimental approaches into standardised disease-modifying treatments capable of restoring disc function, alleviating chronic pain, and improving patient quality of life [2,120,121,122].

## 14. Conclusions

MSC-based therapy represents one of the most promising regenerative strategies for the treatment of intervertebral disc degeneration, offering the potential to move clinical management beyond symptomatic relief toward disease-modifying biological repair. Experimental studies have consistently demonstrated that MSCs can modulate inflammation, restore extracellular matrix homeostasis, promote nucleus pulposus cell survival, and improve disc structure and function through a combination of differentiation and, predominantly, paracrine signalling. Nevertheless, the encouraging results observed in preclinical models have not yet translated into consistent clinical success. This translational gap reflects the combined effects of the hostile intervertebral disc microenvironment, limited cell survival and retention, variability in cell source and manufacturing, heterogeneous clinical protocols, and the absence of standardised biomarkers capable of objectively assessing regenerative outcomes.

Notably, current evidence suggests that the future of regenerative therapy may depend less on identifying a universally superior MSC source than on optimizing the entire therapeutic platform. Advances in biomaterial-assisted delivery systems, hypoxic preconditioning, genetic engineering, extracellular vesicle- and exosome-based therapies, and GMP-compliant manufacturing have the potential to improve treatment reproducibility, biological efficacy, and clinical scalability. Equally important is the integration of quantitative imaging techniques, molecular biomarkers, and precision diagnostic approaches that can facilitate patient selection, enable objective monitoring of biological regeneration, and support personalized therapeutic strategies. While clinical studies have demonstrated encouraging improvements in pain and function, evidence for consistent structural or biological regeneration of the intervertebral disc remains limited. Future trials should therefore evaluate clinical and regenerative endpoints separately and investigate the added value of quantitative imaging and molecular biomarkers alongside patient-reported outcomes.

Ultimately, successful clinical translation will require a multidisciplinary approach that integrates regenerative medicine, biomaterials science, advanced diagnostics, bioengineering, and rigorous clinical investigation. Large multicentre randomised controlled trials employing harmonised manufacturing protocols, standardised delivery strategies, objective imaging and molecular endpoints, and long-term follow-up will be essential to establish the durability, safety, and true disease-modifying potential of MSC-based therapies. As these scientific and technological advances continue to converge, MSC-based regenerative strategies may ultimately evolve from promising experimental interventions into standardised, evidence-based treatments capable of transforming the management of degenerative disc disease and improving long-term patient outcomes.

## Figures and Tables

**Table 1:** Comparison of conventional MSC therapy and MSC-derived exosome therapy for intervertebral disc regeneration.

Characteristic	MSC Therapy	MSC-Derived Exosomes
Therapeutic agent	Viable mesenchymal stromal cells	Cell-free extracellular vesicles (30–150 nm)
Primary mechanism	Paracrine signalling, immunomodulation, and potential differentiation	Paracrine signalling through proteins, lipids, mRNAs and miRNAs
Differentiation potential	Present	Absent
Immunomodulatory activity	High	High
Anti-inflammatory effects	Suppresses inflammatory cytokines and modulates immune responses	Delivers anti-inflammatory proteins and regulatory miRNAs
ECM regeneration	Stimulates aggrecan and collagen II synthesis	Stimulates ECM synthesis through bioactive cargo
Adaptation to host environment	Dynamic; responds continuously to environmental changes	Static; determined by pre-packaged molecular cargo
Tumourigenicity	Low, although potential risks associated with viable cell transplantation remain	Expected to be lower due to the absence of viable proliferating cells; long-term clinical safety remains insufficiently established
Immunogenicity	Generally low	Generally considered low, but dependent on source and product characteristics
Cell survival	Frequently limited within the degenerated disc	Not dependent on cell survival
Manufacturing	Requires cell expansion, viability testing and cryopreservation	Requires isolation, purification and characterisation
Product standardisation	Challenging	More feasible, although not yet standardised
Storage and transport	Complex	Relatively straightforward
Clinical evidence	Multiple clinical studies/trials	Predominantly preclinical evidence; limited clinical evidence
Current limitations	Poor survival, donor variability, senescence, manufacturing complexity	Lack of standardisation, potency assays, pharmacokinetic data and clinical validation
Future perspectives	Optimised cell sources, biomaterials, genetic engineering	Engineered exosomes, targeted miRNA delivery, controlled-release biomaterials

**Table 2:** Representative preclinical studies evaluating cell-based regenerative therapies for intervertebral disc degeneration.

Study	Model	Cell-Type Source	Delivery	Follow-Up	Main Findings	Main Limitation	Reference
Nishimura & Mochida	Rat	Autologous nucleus pulposus cells	Intradiscal implantation	Not reported	Delayed annulus fibrosus degeneration and preservation of nucleus pulposus tissue; first proof-of-concept for cell-based disc regeneration	Used autologous NP cells rather than MSCs; limited translational relevance	[71]
Crevensten et al.	Rat	BM-MSCs	Intradiscal injection	6 weeks	Increased disc height, extracellular matrix production, and cellularity	Small animal model with limited biomechanical similarity to the human spine	[74]
Sakai et al.	Rabbit	BM-MSCs	Intradiscal injection	8 weeks	Differentiation into NP-like cells, increased proteoglycan synthesis, improved disc height and hydration	Short follow-up and limited assessment of long-term regeneration	[75]
Miguélez-Rivera et al.	Rat	MSCs	Intradiscal injection	Not reported	Reduced inflammation and immunomodulatory effects	Functional outcomes and long-term efficacy not fully evaluated	[77]
Canine model study	Dog	MSCs	Intradiscal injection	Not reported	Improved mobility and reduced pain-related behaviour despite limited radiological changes	Limited structural evidence of regeneration and relatively small sample size	[81]

**Table 3:** Clinical studies investigating MSC-based therapies for intervertebral disc degeneration.

Study	Year	Patients	Cell Source †	Treatment Origin	Cell Dose	Study Design	Control Group	Follow-Up	Clinical Outcomes	Adverse Events	Reference
Yoshikawa et al.	2010	2	BM-MSCs	Autologous	NR	Case report	None	2 years	Reduced low-back pain and increased disc water content/MRI signal	No major treatment-related complications reported	[82]
Orozco et al.	2011	10	Culture-expanded BM-MSCs	Autologous	10 ± 5 × 10^6^ cells/disc	Prospective pilot study	None	12 months	Reduced pain and disability; increased disc water content, without recovery of disc height	No treatment-related adverse effects reported	[55]
Pettine et al.	2015	26	Bone marrow concentrate (BMC)	Autologous	Mean 121 × 10^6^ TNC/mL; 2713 CFU-F/mL *	Prospective, open-label pilot study	None	12 months	Significant reductions in VAS and ODI; 8/20 patients with follow-up MRI improved by one modified Pfirrmann grade	No adverse events at aspiration or injection sites; transient post-injection discomfort reported; 2 patients subsequently underwent surgery	[84]
Elabd et al.	2016	5	Hypoxic culture-expanded BM-MSCs	Autologous	15.1–51.6 × 10^6^ cells	Long-term safety and feasibility case series	None	4–6 years	Clinical improvement in most patients; maintenance/reduction of disc protrusion in several cases; no neoplastic changes on follow-up MRI	No treatment-related adverse events reported	[86]
Centeno et al.	2017	33	Culture-expanded BM-MSCs	Autologous	Mean 2.3 × 10^7^ cells (range 1.73 × 10^6^–4.5 × 10^7^)	Prospective registry-based observational study	None	Up to 6 years	Improvements in pain and function; 85% of patients with follow-up MRI showed reduced disc-bulge size	3/33 procedure-related AEs, all pain-related and resolved; no serious AEs; 2 patients subsequently underwent spine surgery	[85]
Amirdelfan et al.	2021	100	STRO-3+ bone marrow-derived mesenchymal precursor cells (MPCs)	Allogeneic	6 × 10^6^ or 18 × 10^6^ cells + HA	Multicentre randomised controlled trial	HA vehicle or saline control	36 months	Improvements in VAS and ODI in MPC groups at several time points; no significant between-group differences in modified Pfirrmann score	Treatment was well-tolerated; no clinical evidence of immune reactions to allogeneic MPCs	[87]

* For Pettine et al., the administered product was bone marrow concentrate rather than culture-expanded MSCs; therefore, the reported BMC composition is provided instead of an inferred MSC dose. † BMC cell counts are not equivalent to MSC doses. Abbreviations: AE, adverse event; BM-MSC, bone marrow-derived mesenchymal stromal cell; BMC, bone marrow concentrate; CFU-F, colony-forming unit-fibroblast; HA, hyaluronic acid; MRI, magnetic resonance imaging; MPC, mesenchymal precursor cell; NR, not reported; ODI, Oswestry Disability Index; TNC, total nucleated cells; VAS, Visual Analogue Scale.

**Table 4:** Major translational barriers limiting the clinical implementation of MSC-based therapy for IVD degeneration and emerging strategies to overcome them.

Translational Barrier	Underlying Cause	Impact on Clinical Translation	Emerging Strategies/ Possible Solutions
Poor MSC survival and retention	Hypoxia, acidic pH, nutrient deprivation, hyperosmolarity, inflammatory microenvironment	Reduced regenerative efficacy and short-term cell persistence	Hypoxic preconditioning, biomaterial-assisted delivery systems, hydrogels, genetic engineering
Cell leakage following intradiscal injection	High intradiscal pressure and annular defects	Loss of therapeutic cells and reduced treatment efficacy	Injectable hydrogels, tissue-engineered scaffolds, optimised delivery techniques
Manufacturing variability	Differences in MSC source, donor characteristics, culture conditions, passage number, and expansion protocols	Poor reproducibility and inconsistent therapeutic outcomes	Standardised Good Manufacturing Practice (GMP)-compliant manufacturing and quality control
Heterogeneity of clinical protocols	Variability in cell dose, injection technique, patient selection, and outcome assessment	Difficult comparison between studies and limited evidence synthesis	Standardised treatment protocols and harmonised clinical trial design
Lack of objective outcome measures	Reliance on patient-reported outcomes (e.g., VAS, ODI)	Limited assessment of true biological regeneration	Quantitative MRI, molecular and biochemical biomarkers, multimodal imaging
Regulatory complexity	Different regulatory frameworks and product classifications across countries	Delayed clinical translation and regulatory approval	International harmonisation of regulatory requirements and standardised potency assays
Limited long-term clinical evidence	Small study populations and short follow-up periods	Uncertain durability of therapeutic efficacy and safety	Large multicentre randomised controlled trials with long-term follow-up

**Table 5:** Emerging strategies to enhance the clinical translation of MSC-based therapy for IVD degeneration.

Emerging Strategy	Mechanism of Action	Potential Advantages	Current Stage of Development
Hypoxic preconditioning	Enhances MSC survival, paracrine activity, and adaptation to the hypoxic disc microenvironment	Improved cell survival, engraftment, and regenerative efficacy	Preclinical
Genetic engineering	Modifies MSCs to overexpress regenerative or anti-inflammatory factors (e.g., GDF-6, TGF-β, BMPs)	Enhanced differentiation, extracellular matrix synthesis, and therapeutic potency	Experimental
Biomaterial-assisted delivery	Injectable hydrogels and tissue-engineered scaffolds improve cell retention and provide mechanical support	Controlled cell delivery, prolonged therapeutic activity, improved tissue regeneration	Preclinical to early clinical
MSC-derived exosomes and extracellular vesicles	Cell-free delivery of bioactive proteins, lipids, and regulatory microRNAs	Reduced immunogenicity, improved safety, scalable manufacturing, and off-the-shelf potential	Early clinical and advanced preclinical
Precision diagnostics	Integration of quantitative MRI, molecular biomarkers, and multimodal patient stratification	Improved patient selection, objective treatment monitoring, and personalized therapy	Emerging
Standardised GMP-compliant manufacturing	Harmonised cell production, quality control, and potency testing	Improved reproducibility, regulatory compliance, and multicentre implementation	Emerging
Artificial intelligence and machine learning	Analysis of imaging and biomarker data for diagnosis and prediction of treatment response	Enhanced patient stratification, outcome prediction, and precision medicine	Emerging

## Data Availability

The data supporting the findings of this study are available from the corresponding author upon reasonable request.
